# Capillary Ionization and Jumps of Capacitive Energy Stored in Mesopores

**DOI:** 10.1021/acs.jpcc.1c00624

**Published:** 2021-04-30

**Authors:** Carolina Cruz, Svyatoslav Kondrat, Enrique Lomba, Alina Ciach

**Affiliations:** † Institute of Physical Chemistry, Polish Academy of Sciences, 44/52, 01-224 Warsaw, Poland; ‡ Max-Planck-Institut für Intelligente Systeme, Heisenbergstraße 3, D-70569 Stuttgart, Germany; § IV. Institut für Theoretische Physik, Universität Stuttgart, Pfaffenwaldring 57, D-70569 Stuttgart, Germany; ∥ Instituto de Química Física Rocasolano, CSIC, Serrano 119, E-28006 Madrid, Spain

## Abstract

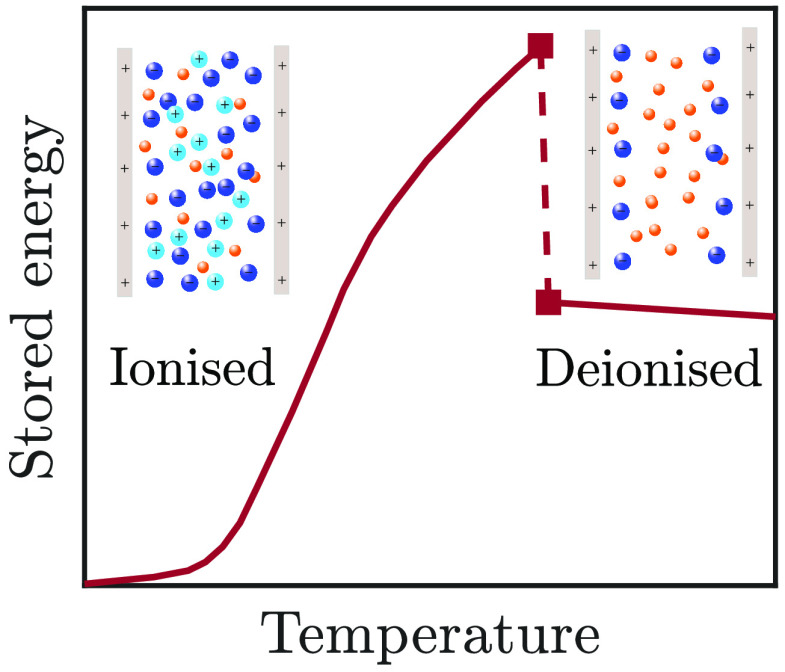

We study ionic liquid–solvent mixtures in slit-shaped nanopores wider than a few ion diameters. Using a continuum theory and generic thermodynamic reasoning, we reveal that such systems can undergo a capillary ionization transition. At this transition, the pores spontaneously ionize or deionize upon infinitesimal changes of temperature, slit width, or voltage. Our calculations show that a voltage applied to a pore may induce a capillary ionization, which—counterintuitively—is followed by a re-entrant deionization as the voltage increases. We find that such ionization transitions produce sharp jumps in the accumulated charge and stored energy, which may find useful applications in energy storage and heat-to-energy conversion.

## Introduction

Ionic liquids (ILs) under confinement play a key role in science and technology, exhibiting remarkable properties and finding applications in energy storage,^1−4^ capacitive deionisation,^5−7^ heat-to-energy conversion,^8−10^ etc. For instance, subnanometer pores filled with an electrolyte provide the highest achievable capacitance^11−13^ and stored energy,^14^ though sluggish dynamics.^15−18^ Electrodes with mesoscale pores avoid a typically poor interpore connectivity of microporous electrodes,^19^ enabling faster charging.^20,21^ Using neat ILs, which have large electrochemical windows,^22−24^ enhances the electrical energy stored in micro- and mesopores.^25^ Unfortunately, neat ILs exhibit slow dynamics; mixing ILs with solvents, such as acetonitrile or water, enhances the IL conductivity^26−29^ and speeds up the charging kinetics.^30^

Previous work has focused on micro- and mesopores filled with IL–solvent mixtures far from phase transitions. However, confined fluids show an exciting physics close to state transformations. A classic example is a capillary condensation,^31,32^ which has numerous practical applications, particularly in determining the pore-size distribution of micro- and mesoporous materials.^33−35^ In this article, we study IL–solvent mixtures liable to phase separation, confined in pores substantially wider than the ion diameter. Using a simple continuum theory and general thermodynamic arguments, we demonstrate that the pores can become spontaneously ionized or deionized in response to small changes in temperature or the applied potential difference. We show that such capillary ionization goes along with abrupt changes in charge and energy storage, which may find practical applications in electrochemical energy storage and generation.

## Model

**Figure 1:**
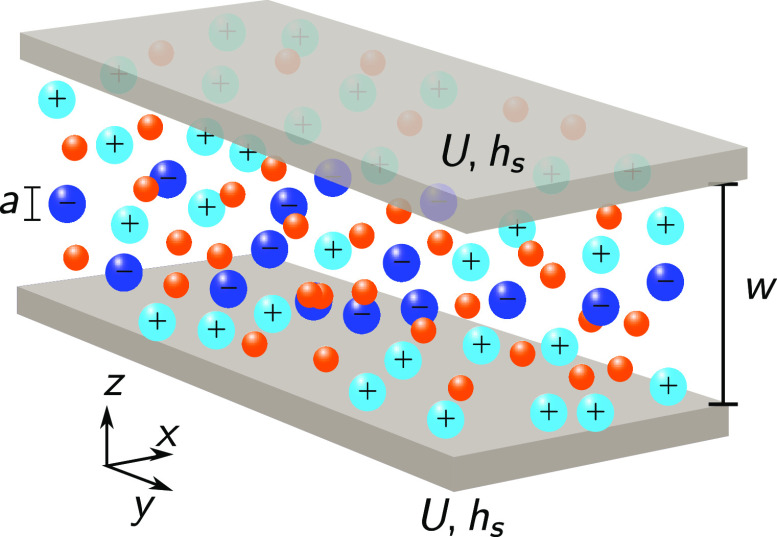
Ionic liquid–solvent mixture in a slit mesopore. The pore walls are separated by a distance *w*. The ion diameter *a* is the same for cations (light blue spheres) and anions (dark blue spheres). Orange spheres represent solvent molecules, modeled implicitly in eq 1. *h*_s_ is the surface field that describes the preference of pore walls for ions or solvent. The potential difference *U* is applied to the pore walls with respect to the bulk electrolyte (not shown). The system is translational invariant in the lateral *x*, *y* directions.

We consider a mixture of a room-temperature ionic liquid and solvent confined into a slit mesopore of a supercapacitor’s electrode, with the potential difference *U* applied to the pore walls with respect to the bulk electrolyte (Figure 1). We model solvent implicitly and assume that the system is translational invariant in the latteral *x*, *y* directions. This system can be described by the grand potential^36,37^
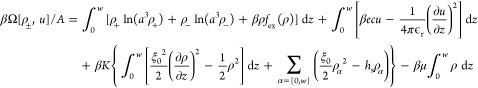
1where β = 1/(*k*_B_*T*) (*k*_B_ is the Boltzmann constant and *T* is temperature), *A* is the surface area, *w* is the slit width, ρ_±_ are the cation and anion densities, and μ is the chemical potential. The charge density is *c* = ρ_+_ – ρ_–_ (in units of the elementary charge *e*) and the total ion density ρ = ρ_+_ + ρ_–_.

The first term in eq 1 is the entropic contribution, which consists of the ideal gas entropy and the free energy density due to excluded volume interactions, *f*_ex_, approximated here by the Carnahan–Starling expression,
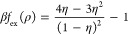
2where η = *πa*^3^ρ/6 is the ion packing fraction and *a* is the ion diameter. The second term in eq 1 is the electrostatic energy, where *u* is the local electrostatic potential and ϵ_r_ is the relative dielectric constant. At room temperature, ϵ_r_ ranges from 80 for water down to about 10 for less polar solvents like alkohols and increases monotonically with increasing temperature.^38−40^ Correspondingly, the Bjerrum length λ_B_ = *e*^2^/(ϵ_r_*k*_B_*T*) acquires a relatively weak dependence on temperature and can increase or decrease with temperature. For simlicity, however, we assume here a temperature-independent Bjerrum length λ_B_ = *a* (corresponding to ϵ_r_ ≈ 80 at room temeprature) and note that the temperature dependence and the choice of ϵ_r_ do not affect our results qualitatively (cf. Figure S5).

The third term in eq 1 arises due to van der Waals interactions, with *K* measuring the strength of the interactions and ξ_0_ their spatial extension.^36^ The parameter *K* sets a temperature (energy) scale, which we express via the critical temperature of a bulk system, as described below. The parameter ξ_0_ is comparable to the molecular dimension and we set it equal to the ion diameter in all calculations.

Ionophilicity *h*_s_ in eq 1 describes the preference of pore walls for ions or solvent. A negative *h*_s_ means that the pore walls favor solvent, while *h*_s_ > 0 means that the walls prefer ions, and we assume that this preference is the same for cations and anions. We focus on ionophilic and weakly ionophobic (*h*_s_ ≈ 0) pore walls and note that even for an “ionophilic” wall, the ion density at the wall can be lower than in the bulk, provided the bulk ion density ρ_*b*_ > *h*_*s*_/ξ_0_.^36^

The equilibrium properties of the system are determined by a minimum of Ω. Minimization of Ω with respect to ρ_±_ and *u* leads to differential equations which we solved numerically (Section S1).

## Results and Discussion

### Bulk System

**Figure 2:**
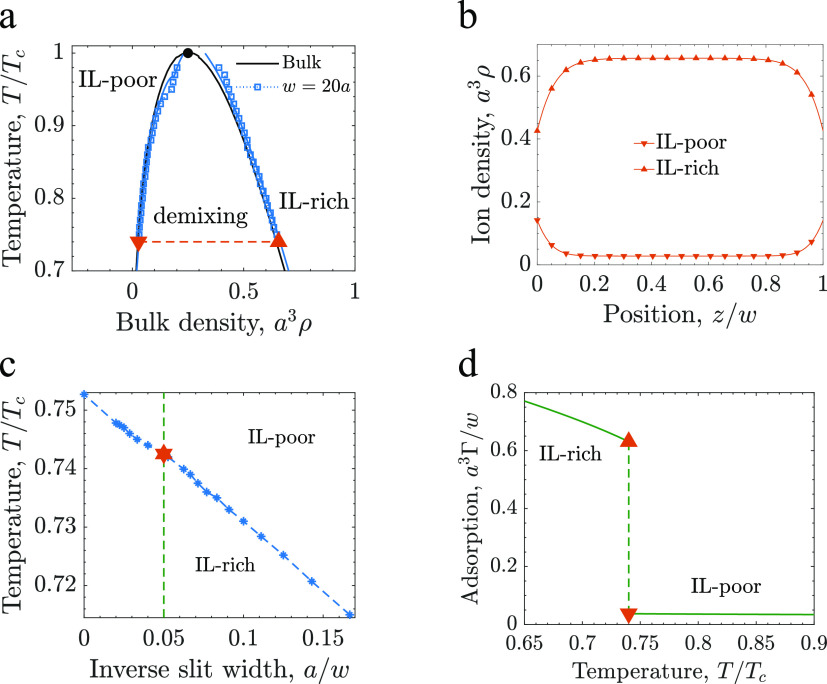
Capillary ionization of uncharged slit mesopores. (a) Phase diagram in the temperature–ion density plane. The solid black line shows the bulk diagram and the circle denotes the critical point. The open squares show the results for a slit of width *w* = 20*a* obtained by numerically minimizing eq 1, and the blue lines show the results obtained by the Kelvin equation, eq 4. The triangles and the dashed line show the temperature *T*/*T*_c_ = 0.74 and the densities at coexistence obtained at the chemical potential μ/*k*_B_*T*_c_ = −4.57; this value of the chemical potential is used in the other panels. Phase diagrams in the chemical potential–temperature and ionophilicity–temperature planes are shown in Figures S1 and S2. (b) In-pore ion density profiles at the coexistence indicated in panel (a). (c) Diagram showing capillary phase transitions in the plane of temperature and inverse slit width. The vertical dash line indicates the slit width used in the other panels. (d) Amount of IL adsorbed in the pore, Γ, as a function of temperature for the slit width *w* = 20*a*. Γ is given by eq 3. In all plots the ionophilicity *a*^3^*h*_s_/ξ_0_ = 0.25, where ξ_0_ is the bare correlation length and *a* the ion diameter.

In a bulk system, that is, outside of the pores, eq 1 simplifies to *βΩ*(ρ̅_b_)/*A* = −*βKρ̅*_b_^2^/2 + ρ̅_b_ ln(ρ̅_b_/2) + *βρ̅*_b_*f*_ex_(ρ*®*_*b*_) – *βμρ̅*_b_, where ρ̅_b_= *a*^3^ρ. The equilibrium condition, ∂Ω_b_/∂ρ̅_b_ = 0, leads to a nonlinear equation, which we solved numerically. The solution reveals the existence of two phases, enriched in ions and solvent, which we call IL-rich and IL-poor phases. Figure 2a (solid black lines) shows that there is a region of temperatures and IL densities, where an IL–solvent mixture separates into the IL-poor and IL-rich phases. This region shrinks for increasing temperature and ends at a critical point *T̅*_c_ = *k*_B_*T*_c_*a*^3^/*K* ≈ 0.09 and ρ_c_*a*^3^ ≈ 0.25.^36^

The bulk phase diagram shown in Figure 2a is typical for IL–solvent mixtures.^41,42^ Various imidazolium tetrafluoroborate ILs in arenes, alkohols, and water have been reported to have critical temperatures in the range from 300 to 400 K and critical IL mole fractions from as low as 0.02 to about 0.125.^42^ A popular 1-hexyl-3-methylimidazolium tetrafluoroborate (C6mim-BF_4_) has the critical temperature *T*_c_ ≈ 326 K and critical mole fraction *x*_c_ ≈ 0.125 in alkohol (C_6_OH) and *T*_c_ ≈ 331 K and *x*_c_ ≈ 0.04 in water.^42^ However, aqueous bistriflimide (TFSI)-based ILs have higher critical concentrations (between 0.2 and 0.3), closer to our model, with the critical temperature ranging from 400 to 420 K.^41^

### Capillary Ionization of Uncharged Pores

The bulk phase behavior of IL–solvent mixtures translates into a similar behavior in uncharged mesopores, where the phase separation region is shifted (symbols in Figure 2a). Examples of the in-pore ion density profiles at coexistence are shown in Figure 2b. The phase diagram in the plane of temperature and inverse slit width consists of a line of first-order phase transitions between the IL-poor and IL-rich phases (Figure 2c). Thus, a phase transition can be induced by changing temperature or slit width. A transition as a function of temperature is illustrated in Figure 2d. This figure shows the amount of an IL adsorbed in the mesopore,
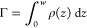
3and demonstrates a *capillary ionization* obtained upon decreasing temperature; that is, that the amount of the IL adsorbed in the pore increases abruptly at the transition as the temperature is decreased.

The location of a capillary ionization transition can be estimated using the Kelvin equation,^43,44^ which in our case reads (Section S2)
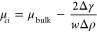
4where μ_bulk_ is the chemical potential of the bulk transition, Δρ is the jump of the IL density at the transition, and Δγ is the difference in the wall–fluid surface tensions of the IL-poor and IL-rich phases. To calculate the surface tensions, we used a semi-infinite system consisting of a single flat electrode. The prediction from eq 4 is shown by blue solid line in Figure 2a and demonstrates good quantitative agreement with the full numerical calculations (see also Figure S1).

**Figure 3:**
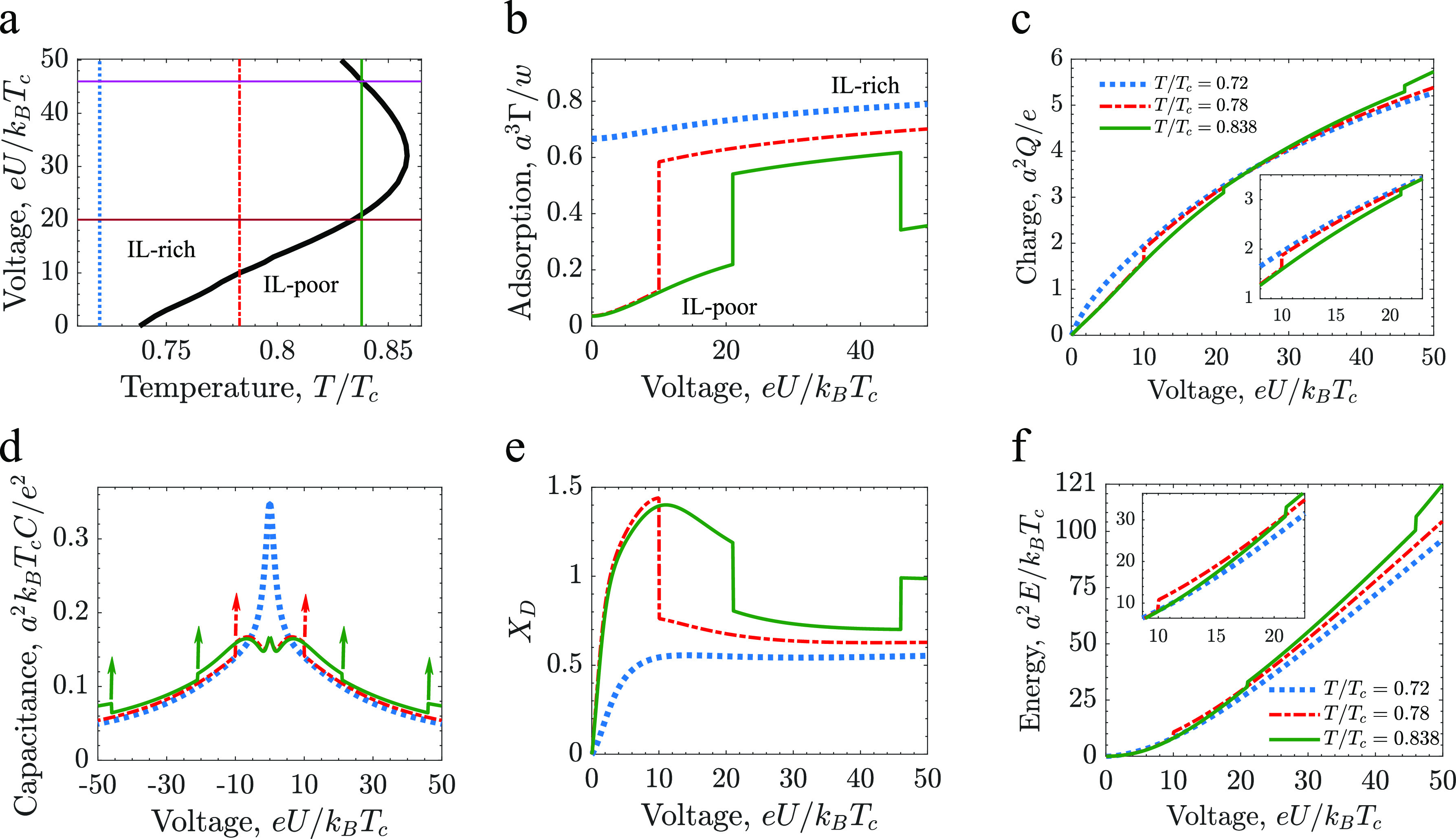
Voltage-induced capillary ionization and chargingof slit mesopores. (a) Phase diagram in the temperature–voltage plane. The thick black line shows a line of first-order transitions betweenthe IL-rich and IL-poor phases. The thin vertical lines indicate temperatures used inthe other panels. The horizontal lines show the values of voltage used inFigure 4. (b) Amount of IL adsorbed in the pore (eq 3), (c) charge accumulated in the pore (eq 5), (d) differential capacitance (eq 6), (e) charging parameter *X*_D_ (eq 1) and (f) energy stored inthe pore (eq 4) as functions of the applied voltage for three values of temperature indicated in (a). The capacitance for*T*/*T*_c_ = 0.78 and *T*/*T*_c_ = 0.838 diverges at the transitions, as schematically denoted by upward pointing arrows. In all plots the pore width *w* = 20*a*, chemical potential μ/*k*_B_*T*_c_ = −4.57 and ionophilicity *a*^3^*h*_s_/ξ_0_ = 0.25, where ξ_0_ is the bare correlation length and *a* isthe ion diameter.For typical values of the ion diameter*a* = 0.7 nm and room temperature for *T*_c_, the various units are thermal voltage *e*/*k*_B_*T*_c_ ≈ 26 mV for voltage, *e*/*a*^2^ ≈ 2 *e* nm^–2^ ≈ 32 μC cm^–2^ for accumulated charge, thermal electric capacitance *e*^2^/(*k*_B_*T*_c_*a*^2^) ≈ 620 μF cm^–2^ for capacitance, and *k*_B_*T*_c_/*a*^2^ ≈ 0.84 mJ cm^–2^ ≈ 0.23 nW cm^–2^ for energy. The color and line codes are the same in panels (b)–(f). Phase diagrams in the chemical potential–temperature plane for a few voltages are shown in Figure S3.. The diagrams for a few other values of the chemical potential are presented in Figure S4 and for a few values of the Bjerrum length in Figure S5. Examples of the total and charge density profiles at the coexistence are shown in Figures S6 and S7.

### Voltage-Induced Capillary Ionization

The black line in Figure 3a shows that the region of the IL-rich phase widens as a voltage is applied to a pore with respect to the bulk electrolyte. The applied potential creates favorable conditions for the counterions to reside inside the pore, which bring along the co-ions (Figure 3e). At high voltages, this region shrinks. The difference between the ion structures near the pore walls in the IL-rich and IL-poor phases decreases for increasing voltage (Figures S6 and S7). Thus, the thermodynamic state becomes determined more and more by the in-pore bulk region, favoring the IL-poor phase at given thermodynamic conditions (Figure S1).

Outside of any transition, ion adsorption (Γ, eq 3), accumulated charge,
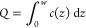
5and the differential capacitance,
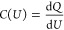
6are all continuous functions of voltage (the blue dashed lines in Figure 3b–d). At low temperatures, the system is in the IL-rich state, characterized by a high ion density, and hence the capacitance has a bell shape (*T*/*T*_c_ = 0.72 in Figure 3d), in accord with numerous studies.^45−53^ At higher temperatures, the IL-poor phase becomes stable and the shape of the capacitance becomes bird-like (*T*/*T*_c_= 0.78 and *T*/*T*_c_ = 0.838 in Figure 3d).^36,54^ The charging mechanisms in these two cases differ significantly, as demonstrated by the charging parameter^55,56^
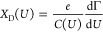
7shown in Figure 3e. In the IL-poor phase at low voltages (*T*/*T*_c_ = 0.72 and *T*/*T*_c_ = 0.78 in Figure 3e), *X*_D_ quickly becomes larger than unity, which means that charging proceeds by adsorption of both co-ions and counterions. In the IL-rich phase (*T*/*T*_c_ = 0.838 in Figure 3e), we have 0 < *X*_D_ < 1 in the whole range of voltages and hence charging is a combination of counterion adsorption and co-ions swapping for counterions.

Applying a voltage to a mesopore can induce a capillary ionization transition (Figure 3a,b). This transition is accompanied by a sharp increase of the charge accumulated in the pore (Figure 3c), which has important consequences for capacitance and energy storage. To analyze charging in this case, we write for the accumulated charge
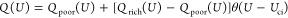
8where *Q*_rich_(*U*) and *Q*_poor_(*U*) are the charge accumulated in the pore in the IL-rich and IL-poor phases, respectively, *U*_ci_ is the transition voltage, and θ(*x*) is the Heaviside step function, equal to unity for *x* > 0 and zero otherwise. Correspondingly, the differential capacitance diverges at the transition, viz., *C*(*U*_ci_) = *C*_poor_(*U*_ci_) + Δ*Q*_ci_δ(*U* – *U*_ci_), where δ(*x*) is the Dirac delta function, *C*_poor_(*U*) = d*Q*_poor_/d*U* is the capacitance in the IL-poor phase, and
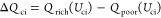
9is the jump of the accumulated charge at the transition voltage *U*_ci_. Thus, the energy stored in the pore,
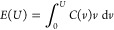
10acquires an additional contribution at the transition, Δ*E*_ci_ = *U*_ci_Δ*Q*_ci_, which appears as a jump in the stored energy (Figure 3f).

**Figure 4:**
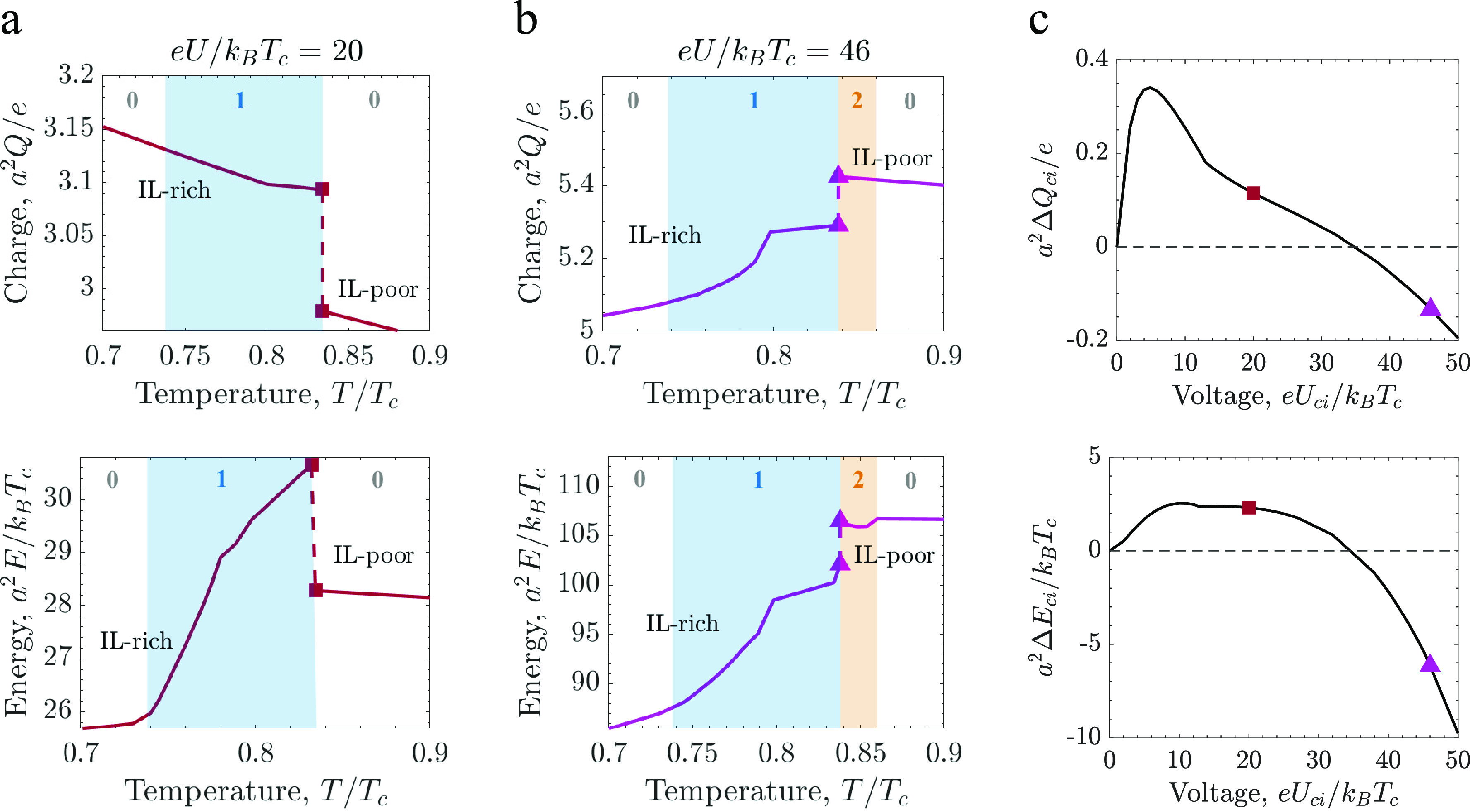
Charge and energy storage in slit mesopores. (a) Accumulated charge (top) and stored energy (bottom) as functions of temperature for appliedvoltage *eU*/*k*_B_*T*_c_ = 20. (b) Same as in (a) but for *eU*/*k*_B_*T*_c_ = 46. The shaded areas show the regions with one and two transitions occurring at voltages below those indicated onthe plots. The number of transitions in each region is also indicated on the plots. (c) Jumps in the accumulated charge (top) and stored energy (bottom) along the transition line as functions of the transition voltage*U*_ci_. The symbols correspond to the jumps shown in (a) and (b). Chemical potential μ/*k*_B_*T*_c_ = −4.57, slit width *w* = 20*a*, and ionophilicity *a*^3^*h*_s_/ξ_0_ = 0.25, where ξ_0_ is the bare correlation length and *a* the ion diameter. For typical values of the ion diameter *a* = 0.7 nm and room temperature for *T*_c_, the various units are thermal voltage *e*/*k*_B_*T*_c_ ≈ 26 mV for voltage, *e*/*a*^2^ ≈ 2 *e* nm^–2^ ≈ 32 μC cm^–2^ for accumulated charge, and*k*_B_*T*_c_/*a*^2^ ≈ 0.84 mJ cm^–2^ ≈ 0.23 nW cm^–2^ for energy. For the phase diagram, seeFigure 3a.

Of course, the jumps of the accumulated charge obtained upon voltage increase are always positive (Figure 3c). Because of the re-entrant behavior, therefore, a jump Δ*Q*_ci_ from the IL-poor to the IL-rich phase (eq 9) is positive for *U* < *U*_b_ and negative for *U* > *U*_b_, where *U*_b_ ≈ 32*k*_B_*T*_c_/*e* is the voltage at which the transition line bends (Figure 3a). In Figure 4a,b, we show examples of the accumulated charge and stored energy density as functions of temperature for *U* < *U*_b_ and *U* > *U*_b_. It is worth noting that there is no monotonous relation between the accumulated charge *Q* (or integral capacitance *C*_I_ = *Q*/*U*) and the stored energy density *E*, as one might expect from the *E* = *C*_I_*U*^2^/2 expression. In particular, an increase in the stored energy may accompany a decrease (Figure 4a) or an increase (Figure 4b) in the accumulated charge. Clearly, the *C*_I_*U*^2^/2 equation is only valid in the linear regime (*Q* ∼ *U*), while the actual charge–voltage relation is more complex.

In Figure 4c, we summarize this behavior by plotting Δ*Q*_ci_ and Δ*E*_ci_ = *U*_ci_Δ*Q*_ci_ along the transition line of Figure 3a. Remarkably, the magnitudes of Δ*Q*_ci_ and Δ*E*_ci_ increase steeply with voltage at high voltages.

## Conclusion

We have studied ionic liquid–solvent mixtures in slit mesopores. We revealed that a pore could become spontaneously ionized as a function of temperature, slit width, or applied voltage, as manifested by jumps in the amount of an ionic liquid adsorbed in the pores. Voltage-induced capillary ionization transitions are exciting as they can be re-entrant and create jumps in the accumulated charge and stored energy density. The possibility to obtain a sharp increase in the stored energy by minute changes of voltage or temperature is spectacular and may find useful technological applications.

Our predictions are based on a simple mean-field model and ideal slit mesopores. In real systems, the locations of the transitions might be shifted by fluctuations, high-density steric repulsions,^57^ and chemical complexity of ion–solvent interactions, all neglected in this work. Furthermore, the distribution of pore sizes and shapes in real electrodes may smear out the jumps at the transitions.^14,58,59^ However, the rapid progress in low-dimensional carbon materials provides hope for engineering highly monodisperse porous structures, e.g., based on MXene^60,61^ or graphene,^62−64^ which would facilitate experimental observation of capillary ionization transitions and enable their application in heat-to-electricity conversion and energy storage.

## Supporting Information Available

Details of the numerical approach, the Kelvin equation, supplementary plots of phase diagrams and of ion and charge densities (PDF)
