# Supplementary material for: Capillary Ionization and Jumps of Capacitive Energy Stored in Mesopores
Source: J Phys Chem C Nanomater Interfaces. 2021 Apr 30;125(19):10243–9. doi: 10.1021/acs.jpcc.1c00624 (PMC8282200; doi:10.1021/acs.jpcc.1c00624)
Supplement: Supplementary file 1 — jp1c00624_si_001.pdf [file jp1c00624_si_001.pdf]

Supplementary Material to: "Capillary Ionisation and Jumps of Capacitive Energy Stored in Mesopores"

Carolina Cruz,[1] Svyatoslav Kondrat,[1,2,3] Enrique Lomba,[4] and Alina Ciach[1]

[1]*Institute of Physical Chemistry, Polish Academy of Sciences, 44/52, 01-224 Warsaw, Poland*

[2]*Max-Planck-Institut für Intelligente Systeme, Heisenbergstraße 3, D-70569 Stuttgart, Germany*

[3]*IV. Institut für Theoretische Physik, Universität Stuttgart, Pfaffenwaldring 57, D-70569 Stuttgart, Germany*

[4]*Instituto de Química Física Rocasolano, CSIC, Serrano 119, E-28006 Madrid, Spain*

S1. NUMERICAL APPROACH

The equilibrium properties of the system are defined by the minimum of the grand potential, Ω. Minimization with respect to u and $\rho_\pm$ gives, in dimensionless form

$$\lambda_D^2 v'' = -c = (1 + \phi/\bar{\rho}_b)\tanh(v) \tag{S1}$$

$$\xi_0^2\,\phi'' + \phi = \bar{T}\left[\ln(1+\phi/\bar{\rho}_b) - \ln(\cosh(v)) + \Delta\mu_{ex}\right] \tag{S2}$$

where $v = eu/k_BT_c$, $\lambda_D = (4\pi\rho_b\lambda_B)^{-1/2}$ is the Debye screening length in bulk electrolyte and ρ_b is the equilibrium ion density (in bulk), $\phi = \bar{\rho} - \bar{\rho}_b (\bar{\rho}_b = a^3\rho_b)$, $\Delta\mu_{ex} = \mu_{ex} - \mu_{ex}^b$ with $\mu_{ex} = \beta\partial f_{ex}/\partial\rho$, $\mu_{ex}^b = \mu_{ex}(z=\infty)$, and $\bar{T} = k_BTa^3/K$ is dimensionless temperature.

The boundary conditions are $v(w) = v(0) = eU/k_BT_c$, where U is the applied voltage at an electrode with respect to bulk, and $\xi_0\phi'(0) - \phi(0) + \bar{h}_s = 0$ and $\xi_0\phi'(w) - \phi(w) - \bar{h}_s = 0$, where $\bar{h}_s = a^3 h_s/\xi_0$.

Equations (S1) and (S2) can be expressed as a reaction-diffusion system that takes the form of semi-linear parabolic partial differential equations as follows:

$$\partial_t q = D\,\partial_{zz}\,q + R(q), \tag{S3}$$

where $q(z,t)$ corresponds to the unknown function, D is the diffusion coefficient, and R accounts for the generation term. Then, by inserting a first order time derivative, we can

write the set of differential equations as follows:

$$\partial_t v = -\lambda_D^2 \partial_{zz} v + \left(1 + \frac{\phi}{\bar{\rho}_b}\right) \tanh(v) \tag{S4}$$

$$\partial_t \phi = -\xi_0^2\, \partial_{zz}\phi - \phi + \bar{T}\Big[\ln\left(1 + \frac{\phi}{\bar{\rho}_b}\right) - \ln(\cosh(v)) + \Delta\mu_{ex}\Big] \tag{S5}$$

By implementing a finite difference scheme forwarded in time and centered in space, we write eqs. (S4) and (S5) in a discrete form:

$$v_m^{n+1} = v_m^n + D_v(v_{m+2}^n - 2\, v_{m+1}^n + v_m^n) + \Delta t\, R_v \tag{S6}$$

$$\phi_m^{n+1} = \phi_m^n + D_\phi(\phi_{m+2}^n - 2\, \phi_{m+1}^n + \phi_m^n) + \Delta t\, R_\phi \tag{S7}$$

where the diffusion coefficients, D_v and D_ϕ, are given by

$$D_v = -\frac{\Delta t\, \lambda_D^2}{\Delta z^2} \tag{S8}$$

$$D_\phi = -\frac{\Delta t\, \xi_0^2}{\Delta z^2} \tag{S9}$$

and Δt and Δz are the temporal and spatial discretization steps. The generation terms, R_u and R_ϕ, are:

$$R_v = \left(1 + \frac{\phi_m^n}{\bar{\rho}_b}\right) \tanh(v_m^n) \tag{S10}$$

$$R_\phi = -\phi_m^n + \bar{T}\Big[\ln\left(1 + \frac{\phi_m^n}{\bar{\rho}_b}\right) - \ln(\cosh(v_m^n)) + \Delta\mu_{ex}\Big] \tag{S11}$$

A. Boundary conditions

The boundary conditions for ϕ are

$$\xi_0\, \phi'(0) - \phi(0) + \bar{h}_s = 0 \tag{S12}$$

$$\xi_0\, \phi'(w) - \phi(w) - \bar{h}_s = 0 \tag{S13}$$

Introducing the temporary derivative and expressing eqs. (S12) and (S13) in a discrete form gives

$$\xi_0 \left(\frac{\phi_2^n - \phi_1^n}{\Delta z}\right) - \phi_1^n + \bar{h}_s = \frac{\phi_1^{n+1} - \phi_1^n}{\Delta t} \tag{S14}$$

$$\xi_0 \left(\frac{\phi_w^n - \phi_{w-1}^n}{\Delta z}\right) - \phi_w^n - \bar{h}_s = \frac{\phi_w^{n+1} - \phi_w^n}{\Delta t} \tag{S15}$$

Then, solving for the node forwarded in time, we have

$$\phi_1^{n+1} = \phi_1^n + \Delta t\Big[\xi_0\left(\frac{\phi_2^n - \phi_1^n}{\Delta z}\right) - \phi_1^n + \bar{h}_s\Big] \tag{S16}$$

$$\phi_w^{n+1} = \phi_w^n + \Delta t\Big[\xi_0\left(\frac{\phi_w^n - \phi_{w-1}^n}{\Delta z}\right) - \phi_w^n - \bar{h}_s\Big] \tag{S17}$$

Likewise, the boundary conditions for the electrostatic potential, v, are

$$v(0) - eU/k_BT_c = 0 \tag{S18}$$

$$v(w) - eU/k_BT_c = 0 \tag{S19}$$

Since there are not spatial derivatives, then

$$v_1^{n+1} = v_w^{n+1} = eU/k_BT_c. \tag{S20}$$

B. Stability

For the model (eqs. (S6) and (S7)) to be stable, the coefficients D_u and D_ϕ must fulfill [1]:

$$D_v \leq 0.25 \tag{S21}$$

$$D_\phi \leq 0.25$$

The values of Δt and Δz have been chosen in such a way that the stability condition is preserved.

S2. KELVIN EQUATION

We consider a mixture of IL and solvent confined in an infinite capillary slit. In the limit of large slit width, w, the total grand potential of the confined phases are the sum of the bulk and surface contributions [2, 3]:

$$\Omega_{\text{rich}} = -p_{\text{rich}} A\, w + 2\gamma_{\text{rich}}(w, \mu) A \tag{S22}$$

$$\Omega_{\text{poor}} = -p_{\text{poor}} A\, w + 2\gamma_{\text{poor}}(w, \mu) A, \tag{S23}$$

where p_{rich} is the pressure of the IL-rich phase and p_{poor} is the pressure of the IL-poor phase at the same chemical potential μ. Additionally, γ_{rich} and γ_{poor} are the surface excess grand potentials of the IL-rich and IL-poor phases, respectively, evaluated at bulk coexistence μ_{bulk} [2–4]. Coexistence of IL-rich and IL-poor phases occurs when $\Omega_{\text{rich}} = \Omega_{\text{poor}}$, then

$$p_{\text{rich}} - p_{\text{poor}} = \frac{2}{w}(\gamma_{\text{rich}} - \gamma_{\text{poor}}) \tag{S24}$$

where $\gamma_{\text{rich}} = \Omega_{\text{rich}} - w\, \Omega_{\text{bulk}}$ and $\gamma_{\text{poor}} = \Omega_{\text{poor}} - w\, \Omega_{\text{bulk}}$. At constant temperature, $dp = \rho\, d\mu$, and by expanding $p_{\text{rich}}(\mu)$ and $p_{\text{poor}}(\mu)$ about p_{bulk}, eq. (S24) can be written as

$$\mu_{\text{bulk}} - \mu = \frac{2}{w} \frac{(\gamma_{\text{rich}} - \gamma_{\text{poor}})}{(\rho_{\text{rich}} - \rho_{\text{poor}})} \tag{S25}$$

This treatment is valid under the assumption that the density profiles are almost constant in the slit [2], that is $\rho(z) \approx \rho_{\text{rich}}$ and $\rho(z) \approx \rho_{\text{poor}}$, where $\rho_{\text{rich}}, \rho_{\text{poor}}$ are the density of the IL-rich and IL-poor phases, respectively.

Confinement shifts the location of the coexistence along a saturation chemical potential curve, μ_{bulk}, to a capillary ionisation curve that occurs along μ_{ci} and which ends at a capillary critical temperature. The phase boundary shift at which capillary ionisation takes place satisfies the macroscopic Kelvin equation [5, 6]

$$\mu_{ci} = \mu_{\text{bulk}} - \frac{2\Delta\gamma}{w\Delta\rho}. \tag{S26}$$

REFERENCES

[1] J. C. Strikwerda, *Finite difference schemes and partial differential equations*, Vol. 88 (Siam, 2004).

[2] A. O. Parry and R. Evans, Universal fluctuation-induced corrections to the Kelvin equation for capillary condensation, J. Phys. A: Math. Gen. **25**, 275 (1992).

[3] R. Evans, Fluids adsorbed in narrow pores: phase equilibria and structure, J. Phys.: Condens. Matter **2**, 8989 (1990).

[4] J. Powles, On the validity of the Kelvin equation, J. Phys. A: Math. Gen. **18**, 1551 (1985).

[5] A. Malijevský and A. O. Parry, Condensation and evaporation transitions in deep capillary grooves, J. Phys.: Condens. Matter **26**, 355003 (2014).

[6] A. Malijevský, A. O. Parry, and M. Pospíšil, Edge contact angle and modified Kelvin equation for condensation in open pores, Phys. Rev. E **96**, 020801 (2017).

SUPPLEMENTARY PLOTS

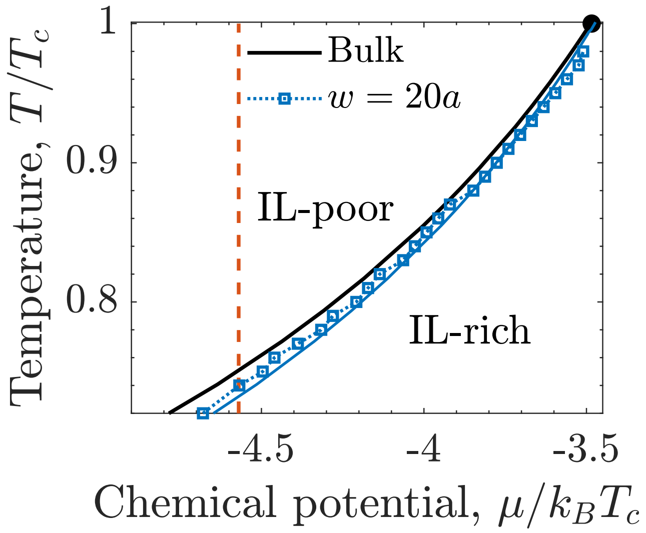

FIG. S1: **Capillary ionisation of non-polarized slit mesopores.** The phase diagram is plotted in the plane of the chemical potential and temperature. The symbols have been obtained by full numerical calculations and the lines corresponds to the results of the Kelvin equation. In confinement, the slit width $w = 20a$, where a is the ion diameter, and the ionophilicity $a^3h_s/\xi_0 = 0.25$, where ξ_0 is the bare correlation length. The vertical line shows the value of the chemical potential $\mu/k_BT_c = -4.57$ used in Fig. 3 and 4 of the main text.

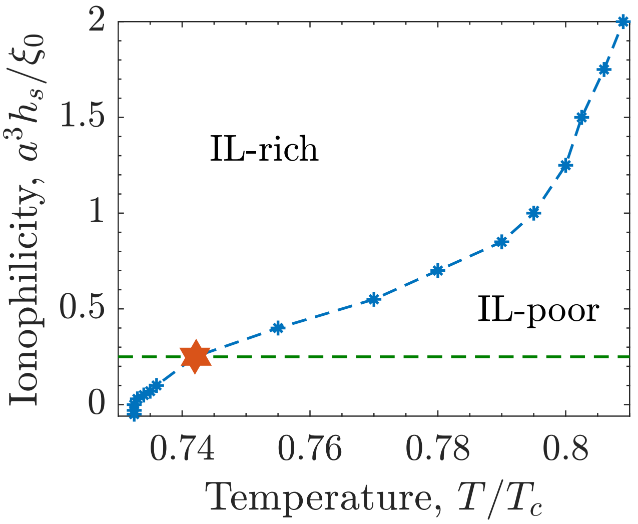

FIG. S2: **Capillary ionisation of non-polarized slit mesopores.** The phase diagram is plotted in the plane of the surface field h_s and temperature. Chemical potential $\mu/k_B T_c = -4.57$ and slit width $w = 20a$. The horizontal line shows the value of h_s used in Fig. 3 and 4 of the main text. The symbols indicate the transition temperature for this value of h (see Fig. 2b,d).

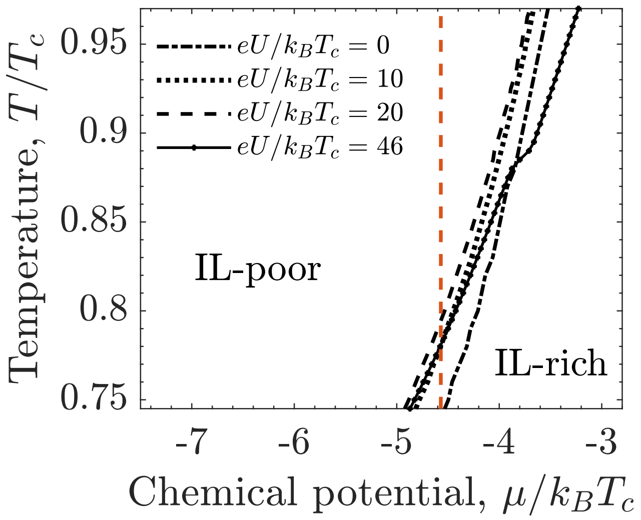

FIG. S3: **Capillary ionisation of slit mesopores under applied voltages.** Phase diagrams are plotted in the (μ, T) plane. The slit width $w = 20a$, where a is the ion diameter, and the ionophilicity $a^3h_s/\xi_0 = 0.25$, where ξ_0 is the bare correlation length. The vertical line shows the value of the chemical potential $\mu/k_BT_c = -4.57$ used in Fig. 3 and 4 of the main text.

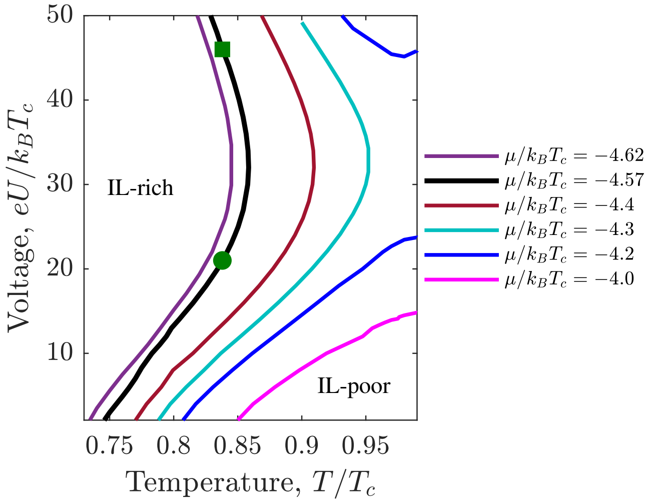

FIG. S4: **Voltage-induced capillary ionisation and de-ionisation of slit mesopores**. Phase diagrams in the temperature-voltage plane showing first-order transitions between the IL-rich and IL-poor phases for a few values of the chemical potential. The black thick line corresponds to the chemical potential $\mu/k_BT_c = -4.57$ of Fig. 3a. The pore width $w = 20a$ and ionophilicity $a^3h_s/\xi_0 = 0.25$, where ξ_0 is the bare correlation length and a the ion diameter. At high temperatures, locating the transitions appears problematic within our numerical approach. The total and charge density profiles at coexistence are shown in Fig. S6 and S7 for the values of temperature and potential difference marked on the plot by symbols.

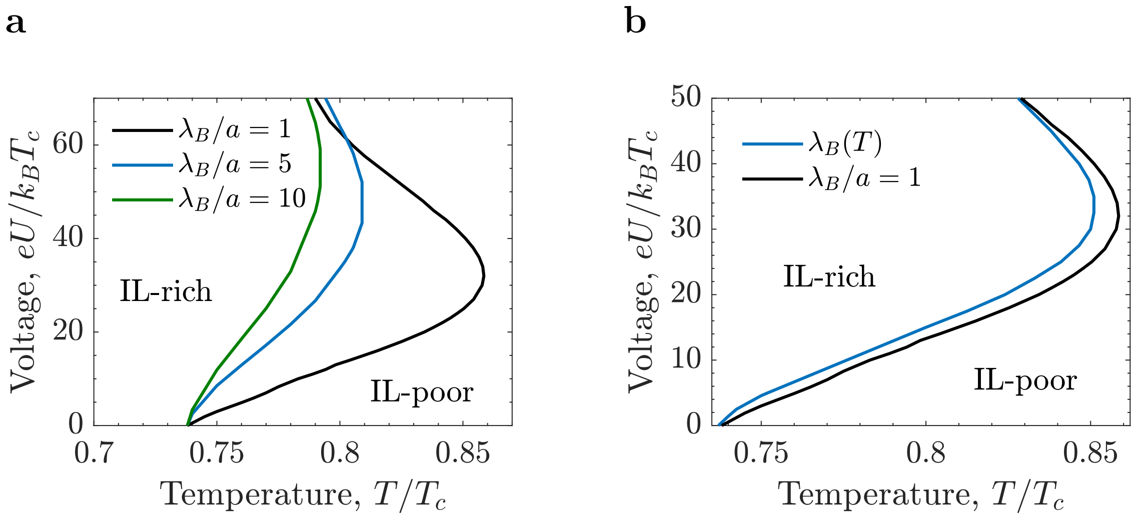

FIG. S5: **Effect of Bjerrum length on voltage-induced capillary ionisation transitions.** (**a**) Phase diagrams in the temperature-voltage plane showing the locations of first-order transitions between the IL-rich and IL-poor phases for a few values of the Bjerrum length λ_B expressed in terms of the ion diameter a. (**b**) Phase diagrams for the Bjerrum length $\lambda_B/a = 1$ and for a temperature-dependent Bjerrum length $\lambda_B(T) = \lambda_B^c(T_c/T)$, where λ_B^c $(= a)$ is the Bjerrum length at the critical temperature T_c. This temperature dependence of $\lambda_B(T)$ means that ϵ_r is temperature independent.

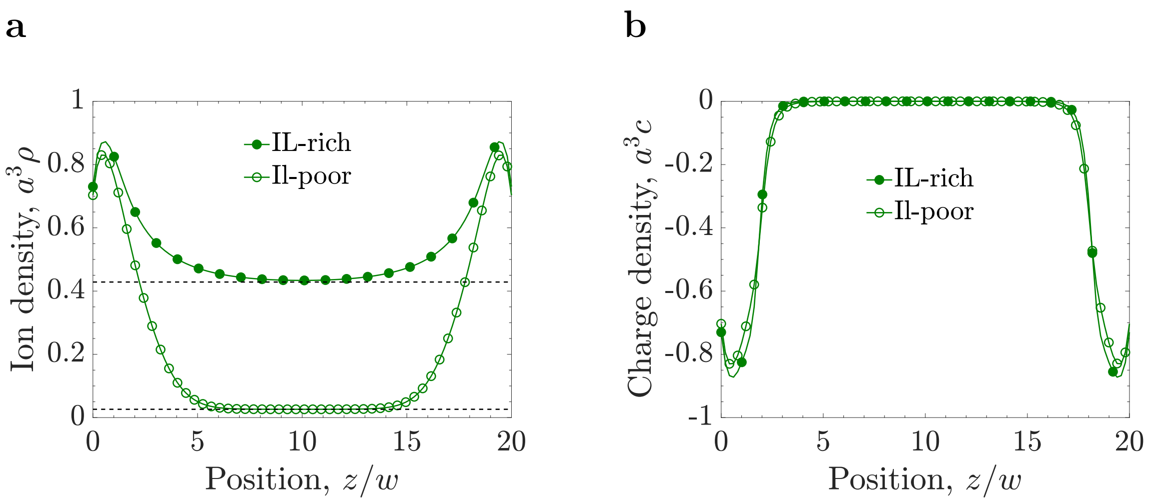

FIG. S6: **Structure of IL-rich and IL-poor phases at coexistence for $eU/k_BT_c = 21$.** (**a**) Total ion density and (**b**) charge density inside a mesopore for $\mu/k_BT_c = -4.57$, $T/T_c = 0.838$ and applied potential difference $eU/k_BT_c = 21$. Slit width $w = 20a$ and ionophilicity $a^3h_s/\xi_0 = 0.25$, where ξ_0 is the bare correlation length and a the ion diameter. The dashed horizontal lines show the bulk values in the corresponding phases.

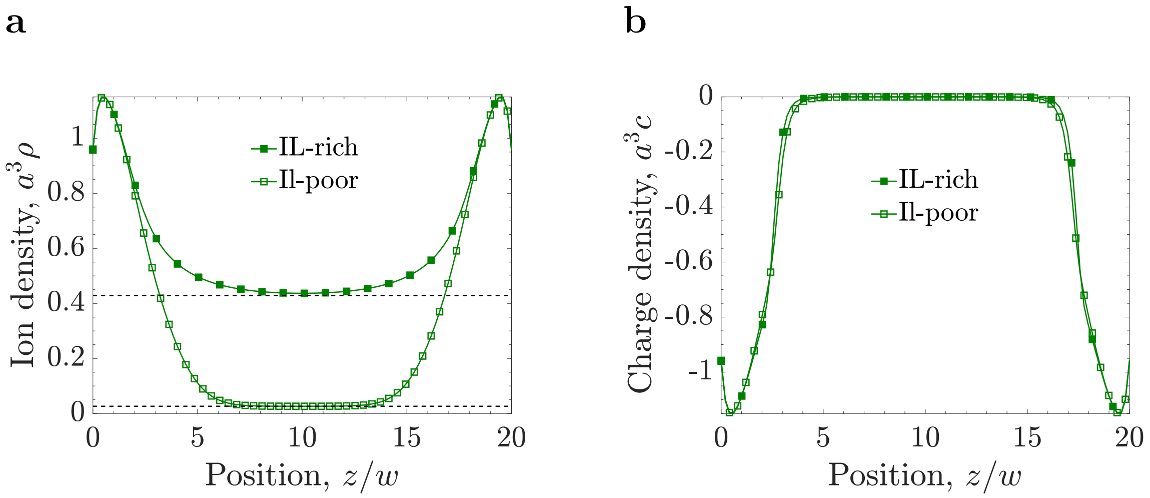

FIG. S7: **Structure of IL-rich and IL-poor phases at coexistence for $eU/k_BT_c = 46$.** (**a**) Total ion density and (**b**) charge density inside a mesopore for $\mu/k_BT_c = -4.57$, $T/T_c = 0.838$ and applied potential difference $eU/k_BT_c = 46$. Slit width $w = 20a$ and ionophilicity $a^3h_s/\xi_0 = 0.25$, where ξ_0 is the bare correlation length and a the ion diameter. The dashed horizontal lines show the bulk values in the corresponding phases.
